# A High-Concentrate Diet Induced Milk Fat Decline via Glucagon-Mediated Activation of AMP-Activated Protein Kinase in Dairy Cows

**DOI:** 10.1038/srep44217

**Published:** 2017-03-13

**Authors:** Lin Li, Yang Cao, Zhenglu Xie, Yuanshu Zhang

**Affiliations:** 1 Key Laboratory of Animal Physiology and Biochemistry, Ministry of Agriculture, Nanjing Agricultural University, China

## Abstract

Dairy cows are often fed a high-concentrate (HC) diet to meet lactation demands; however, long-term concentrate feeding is unhealthy and decreases milk fat. Therefore, we investigated the effects of liver lipid metabolism on milk fat synthesis. Ten lactating Holstein cows were assigned randomly into HC and LC (low-concentrate) diet groups. After 20 weeks of feeding, milk fat declined, and lipopolysaccharide levels in the jugular, portal, and hepatic veins increased in the HC group. Liver consumption and release of nonesterified fatty acid (NEFA) into the bloodstream also decreased. AMP-activated protein kinase alpha (AMPKα) was up-regulated significantly in the livers of the HC-fed cows. The HC diet also up-regulated the expression of the transcription factor peroxisome proliferator-activated receptor α (PPARα) and its downstream targets involved in fatty acid oxidation, including carnitine palmitoyltransferase-1,2 (CPT-1, CPT-2), liver-fatty acid-binding protein (L-FABP), and acyl-CoA oxidase (ACO). The HC diet increased blood glucagon (GC) levels, and liver glucagon receptor (GCGR) expression was elevated. Cumulatively, a long-term HC diet decreased plasma concentrations of NEFA via the GC/GCGR-AMPK-PPARα signalling pathway and reduced their synthesis in the liver. The decreased NEFA concentration in the blood during HC feeding may explain the decline in the milk fat of lactating cows.

In the dairy industry, it is currently common practice to feed a high-concentrate (HC) diet to lactating cows to meet their energy requirements and support high milk production. However, long-term HC feeding is harmful to the health of ruminants and leads to a decrease in milk quality^1^. It has been reported that the feeding of HC diets to lactating cows results in the release of lipopolysaccharide (LPS) from the rumen or hindgut^2^,^3^. Previous studies have shown that free LPS can translocate into the bloodstream from the digestive tract under conditions of high permeability and after injury to the liver tissue^4^. In addition, LPS challenge can induce hepatic oxidative injury by changing the glutathione (GSH) and superoxide dismutase (SOD) enzyme activities^5^,^6^.

Milk fat is an important nutritional ingredient of milk that is beneficial to human health. However, long-term feeding with an HC diet induces a reduction in milk fat^7^. Triglycerides (TG) are the main component of milk fat and are synthesized using fatty acids and α-glycerophosphate in mammary epithelial cells^8^. The uptake of nonesterified fatty acid (NEFA) components by mammary glands is affected by their concentrations in the blood. Previous studies have shown that with an increasing NEFA content in the blood, the absorbed quantity applied to milk fat synthesis was also elevated in mammary cells^9^. Therefore, the substrate precursor of NEFA plays a crucial physiological role in milk fat synthesis. Nutrients required for milk synthesis must be transported from the rumen and gut to the liver to undergo metabolic conversion. In ruminants, the liver is the major site of gluconeogenesis and lipid metabolism, which provides the substrate precursors to the mammary gland for milk production. Liver lipid synthesis and lipidolysis rely on the absorption and utilization of NEFA in the blood^10^,^11^. NEFA are transported through the hepatic portal vein into the liver, where they are metabolized. Then, they exit the liver through the hepatic vein, where they are taken up into the blood.

Several studies have noted that an HC diet leads to a decrease in milk fat. In addition, our previous study showed that the expression profiles of genes involved in the inflammatory response and lipid metabolism in the liver were altered significantly in ruminants after feeding with an HC diet^12^. However, the mechanism of the relationship between liver lipid metabolism and milk fat depression is largely unknown in ruminants fed HC diets for long periods of time. Therefore, the objective of this study was to investigate the potential mechanisms in the liver that contribute to the input of substrate precursors to the mammary gland after feeding an HC diet to lactating cows.

## Results

### There were no differences in the plasma biochemical parameters of the two groups of cows before the experiment

To confirm whether the two groups of cows had the same genetic background, we quantified the plasma biochemical parameters of the dairy cows before the experiment. The concentrations of total protein, albumin, globulin, glutamic-oxaloacetic transaminase (GOT), glutamic-pyruvic transaminase (GPT), alkaline phosphatase (ALP), lactic dehydrogenase (LDH), glucose, total cholesterol, high-density lipoprotein (HDL), low-density lipoprotein (LDL), and TG in the plasma samples from the two groups of cows were not different (Table 1).

### The milk yield and milk composition in the lactating cows fed the low-concentrate (LC) and HC diets

Different diets had no influence on the dry matter intake (DMI) of cows. However, the milk protein and fat content in the HC cows were significantly lower than those of the LC cows (*p* < 0.05). In addition, within 20 weeks of treatment, the milk yield and lactose were higher in the HC cows than that in the LC cows (Table 2).

### HC diet increased the concentrations of LPS in the jugular, hepatic and portal vein plasma

The LPS concentrations in the portal and hepatic veins were significantly increased in the HC cows compared with those in the LC group (*p* < 0.05). Additionally, we also investigated the LPS concentration in the jugular vein and found that it was significantly higher in the HC cows compared with the levels in the LC cows (*p* < 0.05, Table 3).

### An HC diet increased the consumption of TG and NEFA in the liver

**Figure 1:**
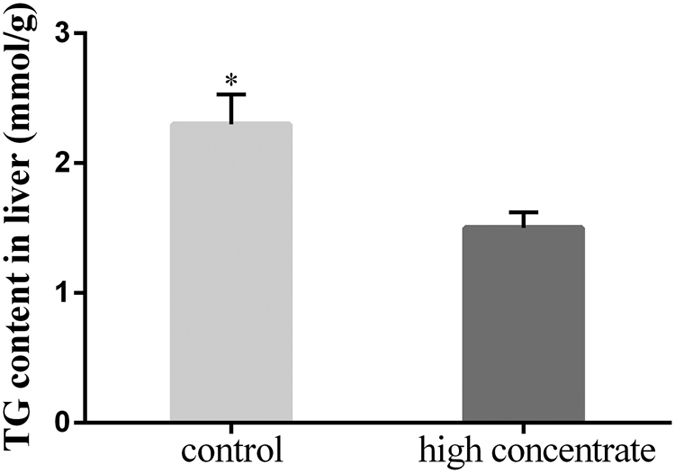
Effect of an HC diet on the TG contents in the livers of lactating cows. Quantification of the liver TG content by biochemical analysis. Data are presented as the means ± SEM (n = 5/group). *p < 0.05 indicates statistically significant differences when compared with the control group.

We next examined nutrition substances in the plasma obtained from the hepatic and portal veins of both treatment groups. We calculated the ratio of portal vein levels:hepatic vein levels (H-P). If H-P > 0, it indicated that more nutrition substances were produced in the liver than entered the blood. Conversely, if H-P < 0, it indicated that nutrition substances were consumed in the liver. Our measurements showed that TG (*p* < 0.01) and NEFA (*p* < 0.05) were significantly lower in HC cows when compared with LC cows. The total cholesterol was consumed in the livers of both HC and LC cows. These findings suggested that more milk fat precursors were consumed in the livers of HC cows (Table 4). In addition, we observed that the liver TG content was significantly lower in the HC cows (*p* < 0.05). This measurement indicates that the TG content was decreased in the liver (Fig. 1).

### HC diet treatment regulated key enzymes required for lipid metabolism in the livers of dairy cows

**Figure 2:**
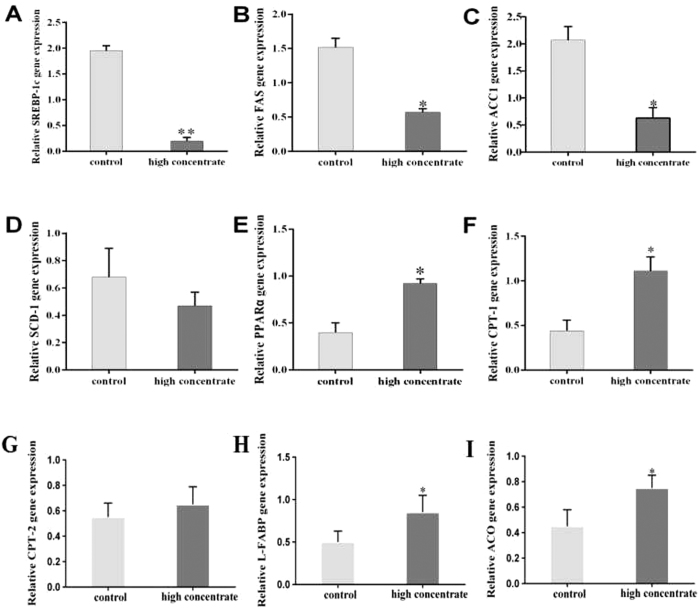
Effects of an HC diet on liver lipid metabolism in lactating cows. (**A**–**D**) The lipid synthesis genes involved in sterol regulatory element-binding protein-1c (SREBP-1c, **A**), fatty acid synthetase (FAS, **B**), acetyl-CoA carboxylase 1 (ACC1, **C**), and stearoyl-CoA desaturase 1 (SCD-1, **D**) were measured in the liver tissue. (**E**–**I**) The lipid catabolism genes involved in peroxisome proliferator-activated receptor α (PPARα, **E**), carnitine palmitoyl transferase-1 (CPT-1, **F**), carnitine palmitoyl transferase-2 (CPT-2, **G**), liver-fatty acid-binding protein (L-FABP, **H**) and acyl-CoA oxidase (ACO, **I**) were measured in the liver tissue. GAPDH was used as the control. The experiments were repeated three times. Data are presented as the means ± SEM (n = 5/group). *p < 0.05 indicates statistically significant differences when compared with the control group.

Sterol regulatory element-binding protein-1c (SREBP-1c) is a key regulator of intracellular lipid metabolism, including uptake and synthesis in the liver. Therefore, we examined the expression of SREBP-1c mRNA and some of its known downstream targets in HC and LC cows (Fig. 2A–D). We found that SREBP-1c expression in the HC cows was significantly lower than in the LC cows (*p* < 0.05, Fig. 2A). The expression levels of downstream targets of SREBP-1c, such as stearoyl-CoA desaturase 1 (SCD-1), acetyl-CoA carboxylase 1 (ACC1) and fatty acid synthetase (FAS), were also decreased in the HC cows (Fig. 2B–D). In particular, FAS and ACC1 expression levels in the HC cows were significantly lower than in the LC cows (*p* < 0.05). Peroxisome proliferator-activated receptor α (PPARα) is a key transcription factor that controls intracellular lipid oxidation, likely by regulating carnitine palmitoyltransferase-1 (CPT-1), carnitine palmitoyltransferase-2 (CPT-2), liver-fatty acid binding protein (L-FABP), and acyl-CoA oxidase (ACO), enzymes required for NEFA oxidation in the liver. We found that the mRNA expression levels of PPARα, CPT-1, L-FABP and ACO were significantly increased in the HC cows compared with the LC cows (*p* < 0.05). However, CPT-2 expression in the HC cows was not significantly different from that in the LC controls (Fig. 2E–I).

### HC diet treatment modulated the AMPK-PPARα signalling pathway

**Figure 3:**
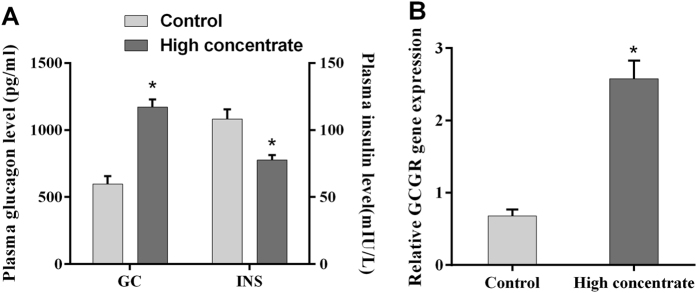
Effects of an HC diet treatment on plasma hormone levels and mRNA expression of the glucagon receptor (GCGR) in the livers of lactating dairy cows. (**A**) Quantification of blood GC and INS levels by ELISA. (**B**) The GCGR mRNA expression level was measured by quantitative PCR. GAPDH was used as the control. The experiments were repeated three times. Data are presented as the means ± SEM (n = 5/group). *p < 0.05 indicates statistically significant differences when compared with the control group.

**Figure 4:**
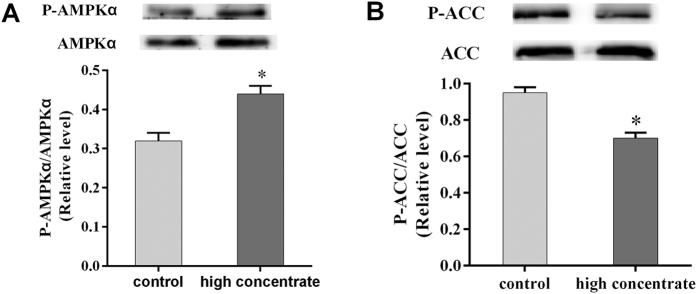
Effects of an HC diet on the expression of phosphorylated AMP-activated protein kinase alpha (p-AMPKα) and phosphorylated acetyl-CoA carboxylase (p-ACC) in the livers of lactating cows. (**A**) Western blot analysis of p-AMPKα protein expression in the liver after treatment with an HC diet. (**B**) Western blot analysis of p-ACC protein expression in the liver after treatment with an HC diet. The experiments were repeated three times. Data are presented as the means ± SEM (n = 5/group). *p < 0.05 indicates statistically significant differences when compared with the control group.

During the course of our earlier experiments, we observed that glucagon (GC) levels in the plasma were significantly higher in HC cows (*p* < 0.05, Fig. 3A). However, we also found that plasma insulin (INS) was significantly lower in HC cows compared with LC cows (*p* < 0.05, Fig. 3A). To further explore a potential mechanism for how the HC diet regulates the expression of key liver enzymes, we examined the activity of the AMPK signalling pathway. The mRNA expression level of the glucagon receptor (GCGR) was significantly higher in the HC cows compared with the levels in the LC cows (*p* < 0.05, Fig. 3B). Western blotting also revealed that the levels of liver P-AMPKα protein in the HC cows were significantly higher than those in the LC cows (*p* < 0.05, Fig. 4A). These findings suggested that the AMPK signalling pathway was activated following treatment with HC diets. We investigated the level of liver P-ACC protein by Western blotting and found that it was significantly lower in the HC cows compared with the levels in the LC cows (*p* < 0.05, Fig. 4B). These findings are consistent with our previous observation that ACC1 mRNA expression was increased in the HC cows.

### An HC diet increased the expression levels of enzymes involved in lipid metabolism and total antioxidant capacity (TAOC) in the liver

**Figure 5:**
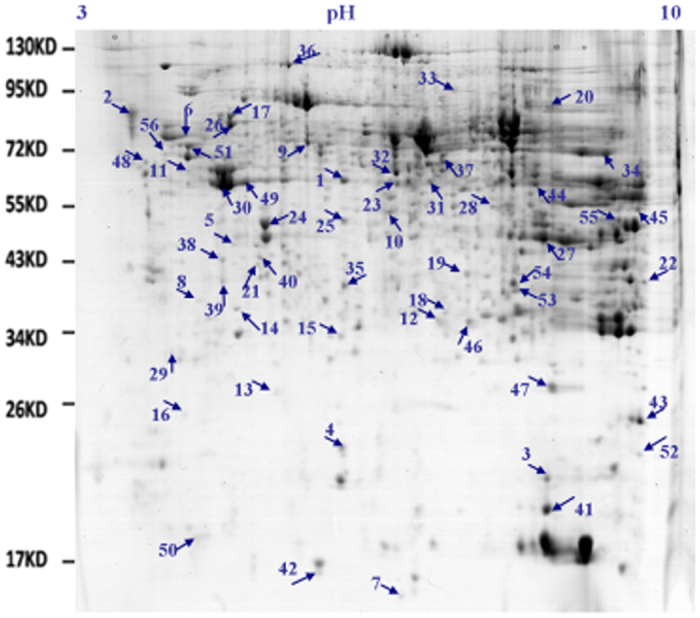
Differentially expressed proteins in the livers of lactating cows fed HC and LC diets by 2-DE analysis. The differentially expressed proteins between HC and LC cows were spotted and numbered. pI, isoelectric point; Mr, molecular mass; n = 5/group.

To investigate the changes in protein expression in the liver tissues of the HC and LC cows, soluble proteins were analysed using the 2-dimensional electrophoresis (2-DE) technique, followed by matrix-assisted laser desorption/ionization–time-of-flight tandem mass spectrometry (MALDI-TOF/TOF) proteomics analysis. Fifty-six differentially expressed proteins were successfully analysed and identified (Fig. 5, Table 5). Three of these proteins, including enoyl-CoA hydratase precursor (ECHS1, spot 41), enoyl-CoA hydratase short chain 1 (spot 42) and 3-ketoacyl-CoA thiolase (ACAA2, spot 45), were up-regulated in the livers of HC cows compared with LC cows. In addition, 3 proteins, including catalase (CAT, spot 1), glutathione s-transferase subunit isoform I (GSTA3, spot 3) and superoxide dismutase [Cu-Zn] (SOD1, spot 7), were also up-regulated in the livers of HC cows compared with LC cows. Taken together, these results suggested that treatment with the HC diet promoted the catabolism of NEFA and TAOC in the livers of HC cows.

## Discussion

In the dairy industry, the practice of feeding HC diets to lactating cows has been applied extensively to increase milk yields, thereby improving cost-efficiency. Although this feeding practice can enhance economic efficiency in the short-term, long-term feeding of HC diets leads to the translocation of LPS from the digestive system into the circulating blood, especially the peripheral circulation system, where it induces a systemic inflammatory response^13^. Previous studies also showed that feeding a diet containing 60% concentrate to lactating goats or cattle elevated blood LPS concentrations and led to LPS translocation and systemic inflammation^13^,^14^,^15^.

As an important defence organ, the liver must process a variety of hazards derived from the portal vein system. The liver is continually exposed to small amounts of LPS translocated from the digestive tract through the mesentery vein directly into the liver via the portal vein^16^. It has been reported that hepatocytes are critical for clearing circulating LPS in the liver^17^,^18^. Therefore, impaired hepatocytes caused by HC diet feeding could contribute to the decreased percentage of LPS clearance in the liver. In the present study, we quantified the plasma biochemical parameters of the dairy cows before the experiment. The biochemical parameters in the plasma samples of the two groups of cows were not different, suggesting that all dairy cows studied had the same genetic background. After 20 weeks of feeding, the concentrations of LPS in the jugular, hepatic and portal vein plasma were markedly increased, indicating that the HC diet induced LPS translocation from the digestive tract into the bloodstream and that the amount of LPS delivered directly into the liver via the portal vein was higher in cows fed an HC diet compared with cows fed an LC diet.

Previous studies have shown that feeding an HC diet to lactating cows can cause an inflammatory response in their livers^19^. In this study, the differentially expressed proteins in the HC- and LC-fed cows were primarily involved in regulating oxidative stress and lipid metabolism. The generation of reactive oxygen species (ROS) is frequently the first detectable response to abiotic or biotic stress in the body. Previous studies have identified various antioxidant enzymes that are involved in ROS metabolism, some of which were differentially expressed in our samples. These enzymes included SOD, GST, and CAT. Cu/Zn-SOD, a product of the SOD1 gene, suppresses metal-catalysed hydroxyl radical production^20^. GST plays an important role in protecting cells from cytotoxic oxidation^21^. In this study, CAT, SOD, and GST were all up-regulated in the HC diet group. Taken together, these results suggested that during feeding with the HC diet, the up-regulated expression of oxidative stress related genes may be triggered by increased LPS translocation into the liver.

Moreover, previous studies have reported that feeding an HC diet could cause a depression of milk fat^22^. In our experiment, decreases in milk fat and milk protein were observed in the HC cows. Therefore, our results were consistent with other studies. However, the mechanism of milk fat depression still requires further study.

NEFA are important precursors in milk fat. Previous studies have reported that feeding an HC diet could cause a depression of milk fat, which was associated with a decline in NEFA in the blood. Increasing the NEFA content in the blood also elevates the absorbed quantity used to synthesise milk fat in mammary cells^23^. Furthermore, we quantified the precursors in milk fat synthesis. The levels of NEFA in the plasma of the HC cows were significantly lower than those in the LC cows. Thus, our results are consistent with other studies. In ruminants, the liver is the major site for gluconeogenesis and lipogenesis, which provides the substrate precursors to the mammary gland for milk production. Therefore, to further study the changes observed in milk fat precursors, we examined the dynamics of NEFA and TG production in the liver by assaying plasma obtained from the hepatic and portal veins. The results suggested that more NEFA and TG were consumed from the livers of HC diet cows compared with cows fed a LC diet. In addition, we found that the liver tissue TG content was significantly decreased in the HC cows, indicating that lipid accumulation in the liver was decreased. However, the relationship between reduced plasma NEFA and the liver warrants further investigation.

SREBPs are transcription factors that activate genes involved in lipogenesis and fatty acid synthesis^24^. SREBP-1c is one member of this family that may regulate many genes involved in lipid synthesis and deposition^25^, such as ACC1, SCD-1, and FAS, which are required for fatty acid synthesis in white adipose tissue, the liver, skeletal muscle, and other tissues^26^. The incidence of fatty liver increased when the expression levels of SREBP-1c and its target genes, FAS and ACC1, were enhanced significantly in bovine hepatocytes^27^. PPARs are involved in the transport of TG in the blood, cellular fatty acid uptake, and mitochondrial beta oxidation^28^. PPARs have three subtypes: PPARα, PPARβ, and PPARγ. PPARα has an important role in the regulation of mitochondrial and peroxisomal fatty acid oxidation in ruminants, including modulation of downstream targets, such as L-FABP, CPT-1, CPT-2 and ACO^29^. In this study, we demonstrated that SREBP-1c expression was significantly decreased in the HC group. Interestingly, the expression profiles of the downstream genes, FAS, SCD, and ACC1, were consistent with the change in SREBP-1c expression. In contrast, the mRNA levels of PPARα and its downstream protein targets, L-FABP, CPT-1, CPT-2 and ACO, were elevated. Moreover, these findings were consistent with ACAA2, ECHS1 and enoyl-CoA hydratase short chain 1 protein up-regulation identified by 2-DE and MALDI-TOF/TOF analysis and the increase in fatty acid oxidation gene mRNA expression in the livers of HC cows. Taken together, these results suggest that the HC diet promotes NEFA catabolism and inhibits NEFA uptake and synthesis by regulating the expression of key liver enzymes in lactating cows. These findings may be useful to explain why milk fat precursor synthesis is decreased when cows are fed a HC diet.

In this study, qRT-PCR was applied to determine the expression of selected liver lipid metabolism genes. Although not used here, the RNA sequencing (RNA-seq) method deserves to be mentioned. This next-generation sequencing-based, high-throughput technique provides gene expression information at the transcriptome levels^30^, compared to qRT-PCR, which analyzes only selected candidate genes. However, in our study, we focused on only a group of genes involved in NEFA metabolism. As shown in Fig. 2, qRT-PCR was sufficient to determine the key enzymes of liver involved in NEFA metabolism. In the following studies, RNA-seq may be applied to identify novel genes and pathways regulated by HC diet in lactating cows.

GC is important for regulating lipid metabolism, in part through its inhibition of fatty acid synthesis in the liver^31^. Previous studies *in vivo* showed that hepatic GC action caused a hepatic energy-depleted state characterized by an increased AMP-to-ATP ratio that was sufficient to activate AMPK^32^. Recent work also extends GC’s novel actions to include AMPK activation of PPARα. This finding is important because PPARα is a transcription factor that is essential for hepatic lipid metabolism^33^,^34^. Previous studies have also shown that the AMPKα signalling pathway could regulate lipid oxidation and synthesis in bovine hepatocytes^35^,^36^. Therefore, the AMPK signalling pathway plays a central role in hepatic lipid metabolism. In the present study, the HC diet increased AMPK protein expression in the liver; the plasma GC levels in the HC cows were also higher. Therefore, we verified that this pathway is activated in the livers of cows that received the HC diet. Furthermore, the AMPK signalling pathway mediates the observed effects on NEFA metabolism by enhancing fatty acid β-oxidation and inhibiting synthesis in the livers of lactating cows.

## Conclusion

**Figure 6:**
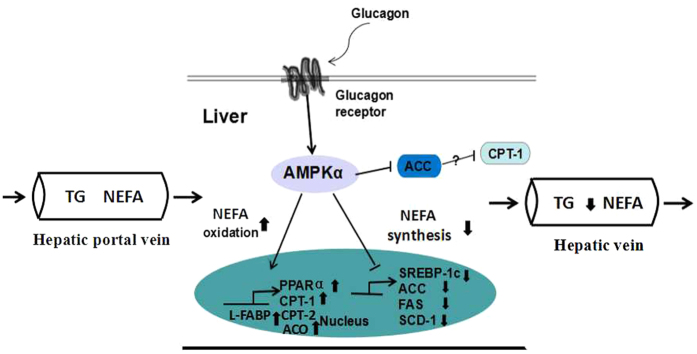
GC activates the phosphorylated AMP-activated protein kinase alpha (p-AMPKα) signalling pathway to regulate lipid metabolism in the liver. An HC diet increased the concentration of GC in the plasma and AMPKα activation. Activated AMPKα increases the expression and transcriptional activity of PPARα, thereby increasing the expression of lipolytic genes, including CPT-1, CPT-2, L-FABP and ACO. AMPKα activation inhibits the expression and transcriptional activity of SREBP-1c, thereby reducing the expression of lipogenic genes, including ACC, FAS, and SCD-1. In addition, activated AMPKα directly phosphorylates and inhibits ACC. Consequently, an HC diet increases lipolysis and reduces lipid synthesis in the livers of lactating cows.

In summary, long-term feeding of an HC diet leads to antioxidant improvement and lipid decreases in the liver. We investigated the effects of the HC diet on NEFA metabolism in the livers of lactating cows and found that NEFA precursors were consumed in the liver and declined in plasma. Furthermore, the plasma GC levels were increased in the HC cows: elevated GC increases AMPKα phosphorylation and activity. Activated AMPKα promotes PPARα expression and transcriptional activity, thereby increasing the expression levels of lipolytic genes. Activated AMPKα inhibits the expression and transcriptional activity of SREBP-1c, thereby down-regulating the expression of the lipogenic genes and reducing lipid synthesis (Fig. 6). Thus, long-term HC diet feeding may lead to the up-regulated expression of lipid oxidation genes and a decrease in the NEFA content in the blood via the GC-activated AMPK/PPARα signalling pathway. However, the decreased NEFA concentration in the blood of cows fed a HC diet may explain the reduction in milk fat in these lactating cows.

## Materials and Methods

### Ethics statement

This animal experiment was reviewed and approved by the Institutional Animal Care and Use Committee of Nanjing Agricultural University. The experiment was performed in accordance with the “Guidelines for Experimental Animals” of the Ministry of Science and Technology (Beijing, China).

### Animals, experimental design and treatment

A total of ten half-sibling, multiparous, mid-lactation Holstein dairy cows (body weight, 489 ± 18 kg, milk yield, 12.24 ± 0.33 kg/d, means ± SEM, 1 to 2 parity) were used in this experiment. All cows were randomly assigned to two groups: one group received an HC diet (Forage: Concentrate = 4:6, HC, n = 5), and the other group received an LC diet (Forage: Concentrate = 6:4, LC, n = 5) as a control for the 20-week experimental period. The ingredients and nutritional compositions of the diets are presented in Table 6. All experimental cows came from the Centre of Experimental Animals at Nanjing Agricultural University and were housed in individual tie-stalls at the Nanjing Agricultural University Experimental Dairy Farm (Nanjing, China). Animals were fed and milked three times daily at 04:00 am, 12:30 am and 19:00 pm and were allowed free access to fresh water. The DMI was measured at each time point. In the first week of the adaptation period, the cows had indwelling hepatic and portal vein catheters placed. After surgery, the cows were observed for 1 week during recovery from the surgery. The animals were looked after for 4 weeks after surgery. Sterilized heparin saline (500 IU/mL) was used to prevent catheter blockage daily at 8-hour intervals until the end of the experiment.

### Milk composition analysis

We collected 200-mL samples of fresh milk into vials with 10 mL of potassium dichromate every week, and the milk fat, protein and lactose concentrations in the samples were analysed using an Integrated Milk-Testing™ Milkoscan 4000 (Foss Electric, Hillerod, Denmark) at the Animal Experiments of Nanjing Weigang Dairy Industry Company.

### Measurement of plasma biochemical parameters

At the beginning of the experiment, plasma was sampled thirty minutes prior to feed delivery using EDTA-containing vacuum tubes from the jugular vein. Blood was centrifuged at 2500 × *g* for 10 min to separate the plasma. The plasma biochemical parameters were measured using a Beckman Kurt AU5800 series automatic biochemical analyser (Beckman Kurt, USA) at the Nanjing Military Region General Hospital (Zhongshan Road, Nanjing, China).

At the end of the experiment, plasma was sampled thirty minutes prior to feed delivery using EDTA-containing vacuum tubes from the jugular, hepatic and portal veins. Blood was centrifuged at 2500 × *g* for 10 min to separate the plasma. The concentrations of TG, NEFA and total cholesterol from portal and hepatic vein plasma were measured using a Beckman Kurt AU5800 series automatic biochemical analyser (Beckman Kurt, USA) at Nanjing Military Region General Hospital (Zhongshan Road, Nanjing, China). The concentrations of INS and GC in the plasma were determined using ELISA kits (Shanghai Enzyme-linked Biotechnology Co. Ltd, Shanghai, China). The detected ranges of the ELISA kits for INS and GC were 0.1–40 mIU/L and 5–1000 pg/mL, respectively. The procedures were performed according to the manufacturer’s instructions.

### Hepatic TG extraction and measurement

After 20 weeks of feeding, liver tissues were obtained by punch biopsy under local anaesthesia. A 100-mg liver sample from each animal was homogenized in 0.5 mL of 1 M NaCl and then extracted with 3 mL of chloroform/methanol (2:1) plus 0.5 mL of 1 M NaCl. The lower phase was collected, dried and resuspended in 1 mL of Triton X-100/methanol (2:1). The TG levels were determined using a Beckman Kurt AU5800 series automatic biochemical analyser (Beckman Kurt, USA) at the Nanjing Military Region General Hospital (Zhongshan Road, Nanjing, China).

### LPS measurements

The LPS concentrations in the jugular, hepatic and portal vein plasma samples were determined using a chromogenic endpoint assay (CE64406, Chinese Horseshoe Crab Reagent Manufactory Co., Ltd., Xiamen, China) with a minimum detection limit of 0.05 EU/mL. The procedures were performed according to the manufacturer’s instructions.

### RNA extraction, cDNA synthesis and quantitative real-time PCR (qRT-PCR)

Total RNA was extracted from liver samples using TRIzol reagent (15596026, Invitrogen, Shanghai, China) according to the manufacturer’s protocol and converted to cDNA using commercial kits (Vazyme, Nanjing, China). All PCR primers were synthesized by Generay Company (Shanghai, China); the primer sequences are listed in Table 7. RT-PCR was performed using the AceQ qPCR SYBR Green Master Mix Kit (Vazyme, Nanjing, China) and the MyiQ2 Real-time PCR System (Bio-Rad, USA) with the following conditions: 95 °C for 2 min, 40 cycles of 95 °C for 15 sec, and 58–60 °C for 30 sec. Glyeraldehyde 3-phosphate dehydrogenase (GAPDH) served as a reference for normalization. The 2^−ΔΔCt^ method was used to analyse the real-time PCR results, and each mRNA level is expressed as the fold change relative to the mean value of the control group.

### Western blotting

Total proteins were extracted from liver samples, and the concentration was determined using a bicinchoninic acid (BCA) assay kit (Beyotime, Shanghai, China). We isolated 30 μg of total protein from each sample, which was subjected to 8–10% SDS-PAGE. The separated proteins were transferred onto polyvinylidene difluoride membranes (Millipore, USA). The blots were incubated with the following Cell Signaling Technology primary antibodies overnight at 4 °C at dilutions of 1:1000 in block: rb-anti-AMP-Activated Protein Kinase alpha (rb-anti-AMPKα, D5A2), rb-anti-Phosphorylated AMP-Activated Protein Kinase alpha (rb-anti-P-AMPKα, T172), rb-anti-acetyl-CoA carboxylase (rb-anti-ACC, #3662S), and rb-anti-Phosphorylated acetyl-CoA carboxylase (rb-anti-P-ACC, #3661S). An rb-anti-GAPDH primary antibody (a531, Bioworld, China, 1:10,000) was also incubated with the blots to provide a reference for normalization. After washing the membranes, an incubation with horseradish peroxidase-conjugated secondary antibody was performed for 2 h at room temperature. The blots were washed, and the signal was detected by enhanced chemiluminescence (ECL) using LumiGlo substrate (Super Signal West Pico Trial Kit, Pierce, USA). The ECL signal was recorded with Substrate (Bio-Rad) using an imaging system (Tanon, Shanghai) and analysed with Quantity One software (Bio-Rad, USA).

### Two-dimensional gel electrophoresis (2-DE)

#### Protein sample preparation

Liver samples were homogenized in an ice-cold buffer (7 M urea, 2 M thiourea, 2% CHAPS, 50 mM DTT, 0.8% CA, 1 mM phenylmethylsulfonyl fluoride (PMSF), Bio-Rad, USA). The homogenates were centrifuged at 15,000 × *g* for 30 min at 4 °C. Total liver protein concentration was determined using an RC-DC^TM^ kit (Bio-Rad, USA).

#### Electrophoresis

2-DE was performed using a 17-cm (nonlinear, pH 3.0–10.0) IPG gel strip (Bio-Rad, USA). Eight hundred fifty micrograms of total liver protein sample was loaded onto the IPG strips (Bio-Rad, USA) using passive rehydration (13 h with 50 V). Isoelectric focusing was performed with a voltage gradient of 250 V for 1 h, 500 V for 1 h, 2000 V for 1 h, and 8000 V for 3 h, followed by holding at 8000 V until 60,000 V-h was reached. Before the second dimension, the IPG strips were first equilibrated for 15 min in 3 mLof equilibration buffer (6 mol/L urea, 50 mmol/LM Tris-HCl pH 8.8, 2% SDS, 30% glycerin, 1% DTT, Bio-Rad, USA) and then underwent a second equilibration for 15 min in the same equilibration buffer except that DTT was replaced by 1% iodoacetamide. Electrophoresis was run initially at 5 W/gel for 30 min followed 15 W/gel via 12.5% SDS-PAGE until the bromophenol blue dye reached the bottom edge of the gel.

#### Statistical analysis

All data are presented as the means ± SEM. Data were tested for normal distribution, and statistical significance was assessed via the independent sample t-test using SPSS version 17.0 for Windows (SPSS Inc., Chicago, IL, USA). Data were considered statistically significant if *p* < 0.05. The numbers of replicates used for statistics are noted in the Tables and Figures.

## Additional Information

**How to cite this article:** Li, L. *et al*. A High-Concentrate Diet Induced Milk Fat Decline via Glucagon-Mediated Activation of AMP-Activated Protein Kinase in Dairy Cows. *Sci. Rep.* **7**, 44217; doi: 10.1038/srep44217 (2017).

**Publisher's note:** Springer Nature remains neutral with regard to jurisdictional claims in published maps and institutional affiliations.

## Figures and Tables

**Table 1:** Plasma biochemical parameters of the two groups of cows before the experiment.

Item	Treatment		p-value
	Control	High concentrate	
Total protein (g/L)	90.9 ± 5.0	88.7 ± 4.7	0.27
Albumin (g/L)	25.0 ± 1.10	26.0 ± 1.3	0.31
Globulin (g/L)	66.0 ± 4.6	63.0 ± 3.8	0.20
GOT (U/L)	64.0 ± 7.82	65.0 ± 6.65	0.12
GPT (U/L)	23.0 ± 3.61	24.0 ± 2.03	0.24
ALP (U/L)	43.04 ± 4.80	39.6 ± 4.70	0.09
LDH (U/L)	923.3 ± 49	901.0 ± 33.5	0.22
Glucose (mmol/L)	3.08 ± 0.17	3.13 ± 0.26	0.11
Total cholesterol (mmol/L)	1.98 ± 0.56	1.83 ± 0.59	0.14
HDL (mmol/L)	1.57 ± 0.41	1.43 ± 0.42	0.21
LDL (mmol/L)	0.13 ± 0.04	0.11 ± 0.01	0.12
Triglyceride (mmol/L)	0.10 ± 0.01	0.11 ± 0.05	0.11

Data are presented as the means ± SEM (n = 5/group).

**Table 2:** Dry matter intake (DMI), milk yield, and milk composition in dairy cows fed low- and high-concentrate diets.

Item	Treatment		p-value
	Control	High concentrate	
DMI, kg/d	16.00 ± 0.28	15.70 ± 1.33	0.82
Milk			
Yield, kg/d	10.93 ± 1.08	14.29 ± 0.41	0.10
Fat content, %	3.94 ± 0.08	3.24 ± 0.12	0.03*
Fat yield, g/d	429.00 ± 32.82	462.50 ± 29.50	0.54
Protein, %	3.40 ± 0.01	3.02 ± 0.05	0.05*
Protein, g/d	372.00 ± 36.76	431.00 ± 19.00	0.32
Lactose,%	4.55 ± 0.19	4.62 ± 0.18	0.81

Data are presented as the means ± SEM (n = 5/group). *p < 0.05 indicates statistically significant differences when compared with the control group.

**Table 3:** Lipopolysaccharide (LPS) concentrations in the jugular, portal, and hepatic veins of lactating cows from treatment and control groups.

LPS (EU/mL)	Control	High concentrate	p-value
Jugular	0.24 ± 0.04	0.62 ± 0.03	0.061*
Hepatic vein	0.21 ± 0.05	0.60 ± 0.03	0.025*
Portal vein	0.30 ± 0.02	0.95 ± 0.05	0.034*

Data are presented as the means ± SEM (n = 5/group). *p < 0.05 indicates statistically significant differences when compared with the control group.

**Table 4:** Effect of an HC diet on plasma indicators in lactating cows.

	Treatment		p-value
	Control	High concentrate	
Hepatic vein (H)			
Triglyceride (mmol/L)	0.11 ± 0.02	0.14 ± 0.03	0.423
Nonesterified fatty acid (mmol/L)	0.81 ± 0.21	0.33 ± 0.02	0.082
Total cholesterol (mmol/L)	1.86 ± 0.40	2.28 ± 0.18	0.401
Portal vein (P)			
Triglyceride (mmol/L)	0.11 ± 0.01	0.22 ± 0.03	0.02*
Nonesterified fatty acid (mmol/L)	0.76 ± 0.20	0.57 ± 0.02	0.38
Total cholesterol (mmol/L)	2.20 ± 0.43	2.62 ± 0.33	0.47
(H-P)			
Triglyceride (mmol/L)	0.00 ± 0.02	−0.08 ± 0.00	0.006**
Nonesterified fatty acid (mmol/L)	0.04 ± 0.05^1^	−0.24 ± 0.04^2^	0.013*
Total cholesterol (mmol/L)	−0.01 ± 0.03	−0.47 ± 0.13	0.072

^1^H-P > 0 represents a lower nutritional substance concentration in the portal vein blood but a higher nutritional substance concentration in the hepatic vein blood, which indicates that the nutritional substances were produced in the liver.

^2^H-P < 0 represents a higher nutritional substance concentration in the portal vein blood but a lower nutritional substance concentration in the hepatic vein blood, which indicates that the nutritional substances were consumed by the liver. Data are presented as the means ± SEM (n = 5/group), *p < 0.05 indicates statistically significant differences when compared with the control group. **p < 0.01 indicates highly statistically significant differences when compared with the control group.

**Table 5:** Identification of differentially expressed liver proteins.

1Spot no.	Protein name	Accession no.	Experimental MW (kDa)/pI	Score	2Fold change
1	Catalase	gi|78369302	60.4/7.06	116	>2.7
2	Endoplasmin precursor	gi|27807263	91.7/4.65	532	>2.3
3	Glutathione S-transferase subunit isoform I	gi|1215748	22.4/8.55	61	>4.0
4	Protein ABHD14B	gi|157428006	23.3/7.0	144	>3.2
5	AKR7A2 protein	gi|151554310	46.6/6.25	123	>2.3
6	Chain A, Structural And Kinetic Analysis Of The Beef Liver Catalase Complexed With Nitric Oxide	gi|332639901	88.6/5.95	529	<0.6
7	Superoxide dismutase [Cu-Zn]	gi|27807109	16.4/7.2	60	>2.1
8	S-formylglutathione hydrolase	gi|115497074	41.3/5.83	102	<0.7
9	Serotransferrin precursor	gi|114326282	74.4/6.6	209	<0.4
10	Reticulocalbin-1	gi|350580184	48.1/7.1	104	>2.1
11	Vimentin	gi|110347570	69.2/5.76	425	>2.0
12	Annexin A4	gi|48374083	39.6/7.3	299	>3.1
13	Nicotinamide N-methyltransferase-like	gi|76635237	32.0/6.42	185	<0.4
14	Nicotinamide N-methyltransferase-like	gi|76635237	36.6/6.24	176	>3.2
15	Thiosulfate sulfurtransferase	gi|29135275	41.9/6.8	530	<0.3
16	Flavin reductase (NADPH)	gi|27806297	28.2/5.8	93	<0.2
17	10-formyltetrahydrofolate Dehydrogenase	gi|296474619	91.7/6.18	812	>2.9
18	Aflatoxin B1 aldehyde reductase member 4	gi|297465355	43.4/7.4	156	<0.2
19	Dihydrodiol dehydrogenase 3	gi|30794344	42.6/7.7	87	<0.6
20	Gastrin Binding Protein-like	gi|3021301	91.6/8.98	179	<0.3
21	3-oxo-5-beta-steroid 4-dehydrogenase	gi|300797655	42.4/6.27	282	<0.4
22	2,4-dienoyl-CoA reductase	gi|115495485	38.0/9.3	94	>2.3
23	Aldehyde dehydrogenase, mitochondrial precursor	gi|115496214	58.8/7.2	187	>2.2
24	dihydrolipoyllysine-residue Succinyltransferase component of 2-oxoglutarate dehydrogenase complex, mitochondrial	gi|115497112	48.1/6.4	103	>2.8
25	Alpha-enolase	gi|87196501	50.2/6.91	107	>2.5
26	Pyruvate carboxylase	gi|28200301	89.7/6.21	623	>2.3
27	Fructose-bisphosphate aldolase B	gi|77735921	45.9/8.45	397	<0.1
28	similar to Succinyl-CoA ligase [GDP-forming] beta-chain, mitochondrial precursor (Succinyl-CoA synthetase, betaG chain) (SCS-betaG) (GTP-specific succinyl-CoA synthetase beta subunit)	gi|146231894	55.0/8.0	170	<0.3
29	Glycerol-3-phosphate dehydrogenase [NAD+], cytoplasmic	gi|78365297	32.6/5.7	66	<0.5
30	S-adenosylmethionine synthase	gi|114052194	59.2/6.15	230	>3.1
31	Glutamate dehydrogenase 1	gi|23306688	60.1/7.5	391	<0.4
32	Glycine amidinotransferase, Mitochondrial precursor	gi|114052741	62.5/7.1	141	>3.4
33	Dimethylglycine dehydrogenase, mitochondrial	gi|329663159	96.8/7.7	117	<0.6
34	Serine hydroxymethyltransferase, Cytosolic	gi|62752042	66.4/9.2	485	<0.4
35	Betaine-homocysteine methyltransferase	gi|86438026	53.6/7.06	591	>2.2
36	Carbamoyl-phosphate synthase [ammonia], mitochondrial	gi|300795597	93.4/6.53	171	>3.1
37	GLUD1 protein	gi|74354891	60.2/7.6	387	>2.6
38	Adenosylhomocysteinase 1-like isoform X1	gi|556763621	44.1/6.19	81	<0.3
39	4-hydroxyphenylpyruvate dioxygenase	gi|62751490	41.5/6.19	192	<0.4
40	Ester hydrolase	gi|114052601	42.9/6.38	69	<0.2
41	Enoyl-CoA hydratase precursor	gi|15982640	20.5/8.7	299	>4.0
42	Enoyl coenzyme A hydratase short chain 1	gi|157057859	15.8/6.68	81	>2.1
43	Mitochondrial enoyl coenzyme A hydratase short chain 1	gi|67944513	24.9/9.4	187	>2.5
44	Delta-1-pyrroline-5-carboxylate dehydrogenase, mitochondrial precursor, Bos taurus	gi|157785571	67.3/8.7	214	<0.6
45	3-ketoacyl-CoA thiolase, mitochondrial	gi|78369436	54.7/9.3	434	>0.4
46	3-hydroxyisobutyrate dehydrogenase, mitochondrial precursor	gi|114052937	34.1/8.1	65	<0.5
47	Eukaryotic translation elongation factor 1 alpha 1 isoform 1	gi|119938328	31.5/8.5	62	<0.3
48	T-complex protein 1 subunit epsilon	gi|274325364	69.3/5.3	129	>2.1
49	60 kDa heat shock protein, mitochondrial	gi|262205483	59.4/6.16	334	>2.1
50	Heat shock protein beta-1	gi|71037405	21.1/6.13	239	<0.4
51	Heat shock cognate 71 kDa protein	gi|76253709	72.2/5.81	216	>2.3
52	protein NipSnap homologue 1	gi|115496626	23.2/9.4	82	>2.2
53	Tubulin beta-2B chain	gi|51491829	42.2/8.37	189	>4.0
54	Tubulin alpha-1D chain	gi|114051854	42.9/8.4	307	>4.0
55	Trifunctional enzyme subunit beta, mitochondrial precursor	gi|27885005	52.7/9.02	185	>2.8
56	Unnamed protein product	gi|428	85.8/5.6	216	>2.2

^1^Numbering corresponds to the 2-DE gel shown in Fig. 1.

^2^Increased (>) or decreased (<) compared with the control group, ≥2.0-fold change in intensity with a *P* -value < 0.05.

**Table 6:** Ingredients and nutritional compositions of the diets.

Ingredient	Percentage (%) of ingredients (dry matter)	
	Control (LC diet)	High-concentrate (HC diet)
Corn silage	30	20
Alfalfa	30	20
Corn	24.3	32
Wheat bran	NA^1^	12.4
Soybean meal	13.5	13
CaHPO_4_	0.85	0.45
Limestone	0	0.8
Salt	0.35	0.35
Premix^2^	1	1
Nutrient levels		
Net energy, MJ/kg	6.36	6.71
Crude protein, %	16.99	16.92
Ether extract, %	3.93	4.07
Neutral detergent fibre, %	36.54	31.45
Acid detergent fibre, %	22.51	17.56
Non-fibre carbohydrate, %	33.76	39.32
Calcium, %	0.88	0.89
Phosphorus, %	0.33	0.46

^1^NA: No action.

^2^Provided per kg of premix: Vitamin A, 6,000 U; Vitamin D, 2,500 U; Vitamin E, 80 mg; Cu, 6.25 mg; Fe, 62.5 mg; Zn, 62.5 mg; Mn, 50 mg; I, 0.125 mg; Co, 0.125 mg; Mo, 0.125 mg.

**Table 7:** Primer sequences used for qRT-PCR analysis of target genes in lactating cows.

Gene	Forward primer (5′-3′); Reverse primer (5′-3′)	Product size (bp)
GAPDH	GGGTCATCATCTCTGCACCT	177
	GGTCATAAGTCCCTCCACGA	
ACC	ACGCAGGCATCAGAAGATTA	179
	GAGGGTTCAGTTCCAGAAAGTA	
FAS	GCACTACCACAACCCAAACCC	161
	CGTTGGAGCCACCGAAGC	
SCD-1	CCGCCCTGAAATGAGAGATG	154
	AGGGCTCCCAAGTGTAACAGAC	
SREBP-1c	CGACTACATCCGCTTCCTTCA	259
	ACTTCCACCGCTGCTACTG	
PPARα	GGAGGTCCGCATCTTCCACT	352
	GCAGCAAATGATAGCAGCCACA	
CPT-1	CCCATGTCCTTGTAATGAGCCAG	254
	AGACTTCGCTGAGCAGTGCCA	
CPT-2	ACGCCGTGAAGTATAACCCT	119
	CCAAAAATCGCTTGTCCCTT	
L-FABP	AAGTACCAAGTCCAGACCCAG	111
	CACGATTTCCGACACCC	
ACO	TAAGCCTTTGCCAGGTATT	189
	ATGGTCCCGTAGGTCAG	
GCGR	TTTCCAGGTGATGTACACGG	141
	TTGAGCATGAAGGACACGAA	
